# Supplementary material for: The effect of SGLT2 inhibitor in patients with type 2 diabetes and atrial fibrillation
Source: PLoS One. 2025 Feb 7;20(2):e0314454. doi: 10.1371/journal.pone.0314454 (PMC11805423; doi:10.1371/journal.pone.0314454)
Supplement: S1 Table — (DOCX) [file pone.0314454.s001.docx]

**S1 Table. The definitions and codes used for each outcome.**

| **Outcomes** | **Logical description and ICD-10 codes** |
| --- | --- |
| Hospitalization for heart failure | Admission with the following ICD-10 codes: I50  Visit ER and diuretics treatment with the following ICD-10 codes: I50 |
| All-cause mortality | All death confirmed by death certificate |
| Cardiovascular mortality | Cardiac death confirmed by death certificate.  Death from ICD-10 codes I00-I99 |
| Stroke | Admission with the following ICD-10 codes: I60, I61, I63, I64, G45, or H34 |
| Myocardial infarction | Admission with the following ICD-10 codes: I21, I22 |
| A composite of major cardiovascular events | All death, myocardial infarction or ischemic stroke |
| Hypoglycemia | Admission with the following ICD-10 codes: E10.0, E11.0, E11.63, E12.0, E13.0, E14.0, E16.0-2 |
