# Supplementary material for: The effect of SGLT2 inhibitor in patients with type 2 diabetes and atrial fibrillation
Source: PLoS One. 2025 Feb 7;20(2):e0314454. doi: 10.1371/journal.pone.0314454 (PMC11805423; doi:10.1371/journal.pone.0314454)
Supplement: Attachment — Submitted filename: 2nd revision response letter.docx [file pone.0314454.s003.docx]

Reviewer #1: 1. The authors have made significant improvements to the presentation of subgroup results. However, the revised statement indicates that the subgroup analysis demonstrates a causal interaction. This finding should also be addressed in the methods section, particularly regarding the interpretation of the p-value for interaction. To further elucidate this observation, I recommend the following article: Brankovic M, Kardys I, Steyerberg EW, et al. "Understanding Interaction (Subgroup) Analysis in Clinical Trials." European Journal of Clinical Investigation. 2019; 49:e13145. <https://doi.org/10.1111/eci.13145>.

We would like to thank you for your valuable comments and suggestions, which have greatly contributed to the improvement of this manuscript. We added/modified the text in the method section and result section as per your suggestion.

Method - Statistical analysis

After propensity score matching, we performed survival analyses to estimate the effect of SGLT2 inhibitors on clinical outcomes. The Kaplan–Meier estimates were performed. The difference between the survival curves was assessed by the log-rank test. A marginal Cox proportional hazards regression models for a cluster in a matched pair were performed. We assessed the consistency of the drug effect on the primary outcome in subgroups. Subgroup analyses were performed to assess whether the effect of SGLT2 inhibitor on clinical outcomes varied across different baseline characteristics. We computed interaction p-values to detect effect modification. An interaction p-value < 0.05 would indicate a statistically significant interaction effect between the treatment and the subgroup variable, suggesting the presence of effect modification, but it is important to note that non-significant interaction p-values do not rule out the possibility of clinically relevant differences in subgroups.^1^ In addition, to evaluate whether the benefit of the SGLT2 inhibitor varied with the follow-up period after the time of initiation, analyses were performed according to the time after initiation of the drug (30, 90, 180 days, 1, and 3 years after the index date). Two-sided p values < 0.05 were considered statistically significant. All analyses were performed with SAS (ver. 9.4; SAS Institute, Cary, NC, USA) and R software (ver. 4.0.3; R Development Core Team, Vienna, Austria).

Result

The effect of the SGLT2 inhibitor on the primary outcome was consistent across all subgroups, indicating that SGLT2 inhibitors were associated with a lower risk of the composite of HF hospitalization or death compared to DPP4 inhibitors, regardless of age, gender, comorbidities, prior history of atherosclerotic CV disease or HF, and the use of beta blocker or renin-angiotension system blocker (all p for interaction>0.05, Fig 3). This suggests that the impact of SGLT2 inhibitor relative to the DPP-4 inhibitor was consistent across the baseline characteristics evaluated. However, this should not be interpreted as definitive evidence of uniform effects across all subgroups. Further studies with more power or focusing on specific subgroups may provide additional insights. Patients with SGLT2 inhibitor demonstrated a significantly lower risk of primary outcome from 90 days after initiating the drug to the third year, as compared with those with DPP4 inhibitor (P<0.05, Table 3).

Reference

1	Brankovic M, Kardys I, Steyerberg EW*, et al.* Understanding of interaction (subgroup) analysis in clinical trials. *European journal of clinical investigation* 2019;49:e13145.
